# KRTCAP2 accelerates malignant progression through modulating tumor cell function and M2 macrophage infiltration in glioma

**DOI:** 10.3389/fimmu.2026.1727168

**Published:** 2026-01-27

**Authors:** Chunmei Zhao, Yijie Sheng, Jianfei Huang, Xudong Wang

**Affiliations:** 1 Department of Laboratory Medicine, Affiliated Hospital of Nantong University, Nantong, Jiangsu, China; 2 Department of Laboratory Medicine, Affiliated Hospital of Nantong University, Medical School of Nantong University, Nantong, Jiangsu, China; 3 Department of Laboratory Medicine, Nantong Hospital of Traditional Chinese Medicine, Nantong, Jiangsu, China; 4 Department of Clinical and Translational Research Center, Affiliated Hospital of Nantong University, Nantong, Jiangsu, China

**Keywords:** cell migration, cell proliferation, immune, KRTCAP2, prognosis

## Abstract

**Background:**

Aberrant glycosylation of proteins plays a critical role in promoting tumor growth and modulating immune responses within the tumor microenvironment. Keratinocyte-associated protein 2 (KRTCAP2) encodes a corresponding protein involved in N-glycosylation, yet its functional and clinical relevance in glioma remains poorly understood. This study aims to elucidate the dual functions of KRTCAP2 in glioma tumorigenesis and immune evasion, and to evaluate its potential as a prognostic biomarker.

**Methods:**

We employed bioinformatics analysis to evaluate KRTCAP2 mRNA expression patterns and immune microenvironment scores in glioma. Multiplex immunohistochemistry (mlHC) was utilized to assess KRTCAP2 protein expression, its association with clinical features, patient prognosis, and immune cell infiltration. To assess the function of KRTCAP2 in regulating glioma cell behaviors and its influence on the tumor immune microenvironment, a set of *in vitro* functional assay were conducted. Additionally, the relationship between KRTCAP2 expression and therapeutic response was examined using the CelIMiner Cross-Database and apoptosis assays.

**Results:**

KRTCAP2 mRNA and protein levels were markedly upregulated in glioma tissues compared to non-tumorous brain tissues. Elevated KRTCAP2 protein expression correlated with adverse clinical characteristics and reduced overall survival. Notably, KRTCAP2 expression showed a significant positive association with the density of infiltrating CD68+ and CD163+ tumor-associated macrophages (TAMs). Depletion of KRTCAP2 significantly inhibited the proliferative, migratory, and invasive capacities of glioma cells, confirming its role as an oncogenic driver. Conversely, KRTCAP2 overexpression promoted these malignant behaviors. Drug sensitivity analysis suggested a role for KRTCAP2 in chemoresistance, with targeted inhibition enhancing glioma cell responsiveness to temozolomide (TMZ).

**Conclusions:**

Our findings identify KRTCAP2 as a novel prognostic biomarker in glioma, with potential utility in predicting immunotherapy response. The study highlights its clinical significance and multifaceted role in shaping an immunosuppressive glioma microenvironment, underscoring KRTCAP2 as a promising therapeutic target.

## Introduction

Glioma, which accounts for about 80% of primary brain tumors, is a highly aggressive cancer originating from glial cells within the central nervous system. It is characterized with high recurrence rates, mortality, and morbidity (1). Glioma cells display malignant biological features such as rapid proliferation, apoptosis resistance, and strong invasiveness, leading to suboptimal clinical outcomes (2). Immunotherapy has recently arisen as a promising approach in oncology, leveraging the body’s immune defenses to identify and eradicate malignant cells. Nevertheless, the microenvironment in glioma is composed of highly heterogeneous cell types that facilitate escape from immune detection and diminish the efficacy of targeted treatments (3). Within this complex tumor microenvironment (TME), tumor-associated macrophages (TAMs) constitute the most abundant population of immune cells. They play a central role in tumor-related inflammation and are critical promoters of tumor progression (4–6).

The Cancer Genome Atlas (TCGA) offers an extensive compilation of clinical and molecular data derived from more than 10,000 tumor patients across 33 cancer types, representing an invaluable resource for large-scale biological and translational research (7). The Chinese Glioma Genome Atlas (CGGA) comprises approximately 2,000 glioma samples with comprehensive clinical annotations, serving as a widely accessible resource for both biological investigation and clinical research (8). Advances in bioinformatics have facilitated the screening of glioma prognostic markers (9–11). Our preliminary TCGA analysis revealed significantly elevated Keratinocyte-associated protein 2 (KRTCAP2) mRNA expression in glioma tissues compared to non-glioma samples, suggesting a potential correlation with clinical outcomes. Additionally, we analyzed KRTCAP2 correlation with clinicopathological features using TCGA and CGGA. KRTCAP2 is situated on human chromosome 1q22 and encodes a protein involved in glycosylation, a process critical to biological functions including cell recognition, immune regulation, and signal transduction (12, 13).

Emerging evidence has identified aberrant glycosylation as a hallmark of cancer (14). This alteration promotes glioma progression and contributes to resistance to temozolomide (15). Furthermore, aberrant glycosylation leads to hyperactivation of aerobic glycolysis, which triggers the release of hexokinase 2—a pivotal glycolytic enzyme—from mitochondria, followed by its interaction with an NF-kB inhibitor. This interaction results in the upregulation of PD-L1 and the establishment of an immunosuppressive tumor microenvironment (16). These modifications also enhance tumor cell adhesion, promote invasive behavior, and facilitate immune evasion, collectively accelerating cancer progression (17–19). Notably, KRTCAP2 has recently been proposed as a prognostic indicator in hepatocellular carcinoma, with possible relevance to immunotherapy efficacy (12). However, its role in glioma progression remains unreported. Thus, exploring KRTCAP2’s function in the glioma immune microenvironment and it’s prognostic significance is critical for advancing therapeutic strategies.

This study sought to investigate the immunological and prognostic significance of KRTCAP2 in glioma. We evaluated the impact of KRTCAP2 expression on patient prognosis and the levels of tumor-infiltrating immune cells. *In vitro* experiments evaluated phenotypic changes in glioma cells following KRTCAP2 downregulation, and we explored the mechanisms through which KRTCAP2 contributes to tumor immune evasion and drug resistance. Our goal was to elucidate KRTCAP2’s role in glioma resistance to immunotherapy and support the development of more effective treatments.

## Materials and methods

### Data source and processing

We obtained uniformly processed RNA-seq data from TCGA (https://portal.gdc.cancer.gov) and Genotype-Tissue Expression database (GTEx, https://www.gtexportal.org/home/) through the UCSC XENA platform (https://xenabrowser.net/datapages/). These databases provided expression matrices for the KRTCAP2 gene along with clinical information from both normal (N = 1157) and tumor (N = 689) tissues. Data preprocessing and normalization were performed using R software (version 4.2.1) (20). We then retrieved clinical data to evaluate whether KRTCAP2 expression is correlated with overall survival (OS), disease-free survival (DFS), and disease-specific survival (DSS) in glioma patients. Additionally, we retrieved genomic data and matched clinical information from the TCGA GBM datasets, along with from the mRNAseq_325 (CGGA 325) and mRNAseq_693 (CGGA 693) datasets available in CGGA (http://www.cgga.org.cn) for further clinical characterization.

### Human specimens

We collected tissue samples from 303 patients with primary glioma and 25 controls with benign brain diseases at the Affiliated Hospital of Nantong University. (March 2013-December 2017). Pathological typing and World Health Organization (WHO) grading were performed in accordance with the 2021 WHO Classification of Tumors of the Central Nervous System (21, 22). This study was approved by the Human Research Ethics Committee of the Affiliated Hospital of Nantong University (Approval number: 2018-K020).

### Tissue microarray construction and fluorescence-based mIHC staining

Formalin-fixed paraffin-embedded glioma tissues were used to construct TMAs with 2mm diametercores (Quick Ray Tissue Microarrayer, UNITMA, Seoul, Korea) (23). Multiplex immunohistochemistry (mIHC) was conducted using the Opal™ 7-Color Manual IHC Kit (NEL810001KT, USA). Tissue microarray sections were first deparaffinized and rehydrated, followed by heat-induced antigen retrieval using an AR6 solution (AR900, PerkinElmer) in a microwave oven. This was followed by incubation with primary antibodies, and a secondary antibody (ARH1001EA; Akoya Biosciences). Fluorescent dyes were added for signal visualization, and multiplex staining was achieved by repeating the above process. Finally, FluoroshieldTM containing DAPI (F6057, Sigma, NY, USA) was used for nucleus staining to facilitate visualization. The antibodies utilized in this experiment are listed below: anti-KRTCAP2 antibody (1:100, orb184599, Biobyt, UK), anti-CD68 antibody (1:1500, 76437S, CST, USA), anti-CD163 antibody (1:200, 93498S, CST, USA).

Slides were scanned with the Vectra 3.0 Imaging System (Akoya Biosciences), and fluorescence intensity was quantified using InForm software (version 26.1, Akoya Biosciences). Positive staining was normalized to the number of nuclei, with scores ranging from 0-100%.

### Cell culture and transfection

Human glioma cell lines U87, T98G, HS683, and U251 were cultured in DMEM/MEM (BasalMedia Technologies) supplemented with 12% fetal bovine serum (Biological Industries) at 37 °C with 5% CO2. KRTCAP2 knockdown plasmids (sh-KRTCAP2-1/2/3), control (sh-NC), overexpression plasmid (pcDNA-KRTCAP2), and control (vector)were synthesized by GeneCopoia. We utilized Lipofectamine 3000 (Invitrogen) to perform the transfection. The shRNA sequences are provided as follows. shNC: TTCTCCGAACGTGTCACGT; sh1:GCTTCGCGCCGTAGTCTTA; sh2:ATGCAGATGTACAGCCGTCAG; sh3:CCTTCAATAATCTGGAGAATC.

### Western blot analysis

Transfected U87 and U251 cells were lysed with RIPA buffer (Beyotime). Protein concentrations were quantified with a BCA protein assay kit (Beyotime). Equal protein amounts were resolved by 10% SDS-PAGE, transferred to nitrocellulose membranes (Millipore), and incubated overnight primary antibodies. Horseradish peroxidase-conjugated secondary antibodies (Abcam) were incubated for 3h at room temperature, and bands were visualized with ECL substrate (Vazyme).

### Cell counting kit-8 assay

Cell proliferation was evaluated using the CCK8 (Dojindo, Japan) according to the manufacturer’s instructions. Transfected cells were cultured for 24, 48, 72, or 96 h, with 10 μL CCK-8 solution added per well and incubated for 3h at 37°C.

### Colony formation

For colony formation assay, 800 cells per well were seeded into 6-well plates and allowed to adhere for 24 hours under standard culture conditions. Following a two-week incubation period, the cells were fixed with 4% paraformaldehyde (PFA) for 45 minutes at room temperature and subsequently washed twice with phosphate-buffered saline (PBS). The fixed cells were then stained with 0.5% crystal violet solution for 1.5 hours, followed by another PBS wash to remove excess stain. Colonies were imaged using a digital imaging system.

### Transwell assay

Cell migration (uncoated chambers) and invasion (Matrigel-coated chambers) were assessed by seeding 5x10^4^ cells/well in serum-free medium. The lower chambers were filled with medium containing 12% FBS. After 48 h, cells were fixed, stained, and quantified.

### Quantitative real-time polymerase chain reaction

KRTCAP2 mRNA expression was measured via qPCR and GAPDH was used as the internal reference. Primers: KRTCAP2 forward (5’-ACTGACTGAACTCATCACGT-3’), reverse (5’-ACATCTCGATGATCGACGGA-3’), GAPDH forward (5’-GGAAATCCCATCACCATCTTC-3’), reverse (5’-TGGACTCCACGACGTACTCAG-3’).

### Immunofluorescence analysis

Cells were fixed in 3.5% paraformaldehyde, permeabilized using 0.1% BSA with 0.5% Triton X-100, blocked, and incubated with anti-KRTCAP2 primary antibody (37°C, 60 min). Secondary antibody incubation (37°C, 60 min) was followed by DAPl counterstaining and fluorescence microscopy.

### Immune cell infiltration analysis

ESTIMATE software (version 1.0.13) was used to compute immune, stromal, and ESTIMATE scores from TCGA RNA-seq data. Spearman’s correlation analyzed associations between scores and KRTCAP2 expression. CIBERSORTx (http://cibersort.stanford.edu/) was employed to quantify the infiltration of 22 immune cell types based on marker gene expression.

### Analysis of apoptosis progression

Following 48 hours of transfection, cells were collected, washed twice with ice-cold phosphate-buffered saline (PBS). Apoptosis was detected using a commercial Annexin V-based Apoptosis Detection Kit (BD Biosciences, USA) according to the manufacturer’s instructions.

### Statistical analysis

Data analysis was conducted using SPSS (version 26.0), and graphs were generated using GraphPad Prism (version 8.3.0). Pearson’s chi-squaremtest assessed associations between KRTCAP2 expression and clinicopathological features. Student’s t-test was applied to compare quantitative data. A two-sided P-value < 0.05 was considered statistically significant.

## Results

### Association between KRTCAP2 mRNA expression and survival in glioma patients

**Figure 1:**
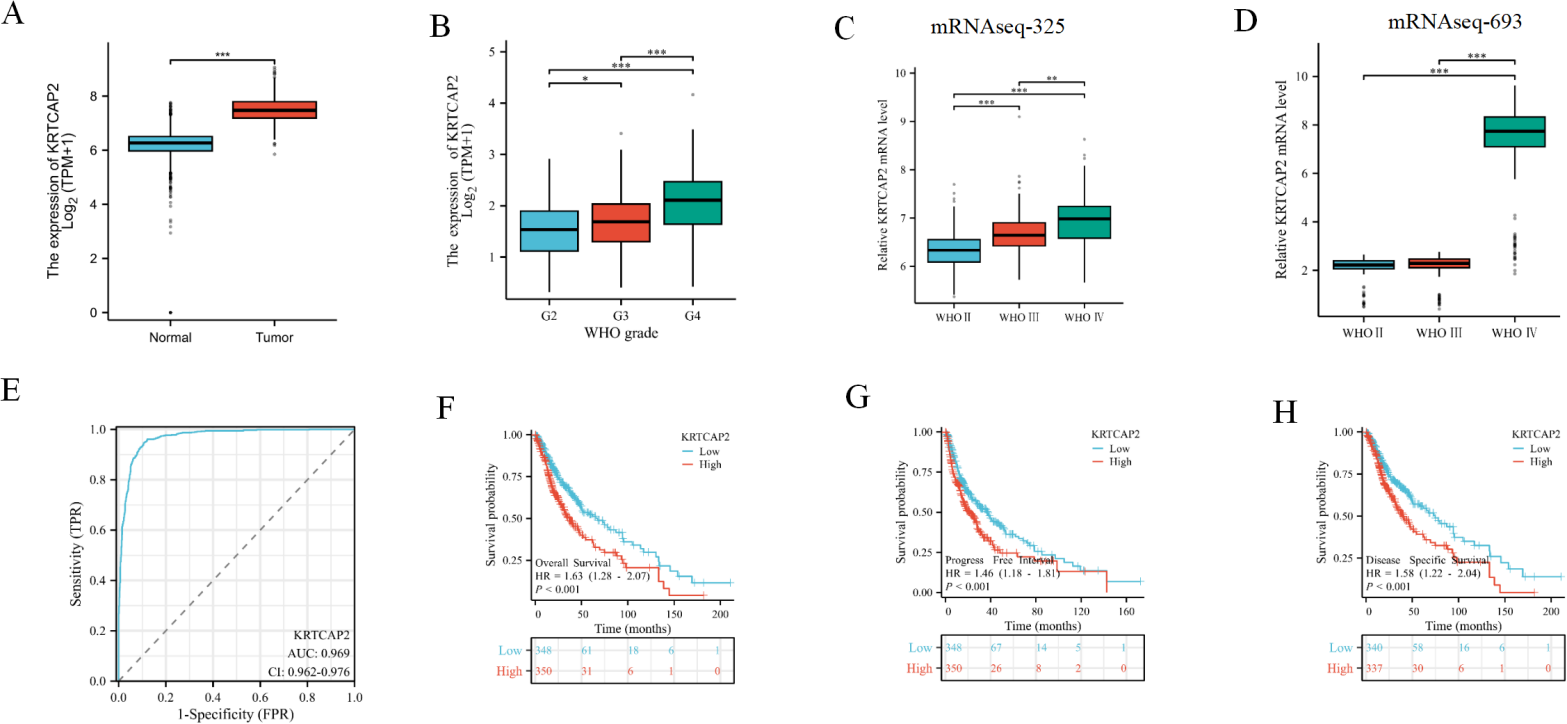
KRTCAP2 mRNA expression in glioma tissues. **(A)** KRTCAP2 expression difference between non-tumor tissue and tumor tissue samples in TCGA and GTEx databases. **(B–D)** KRTCAP2 was significantly increased in high-grade gliomas in the TCGA database and the CGGA 325 dataset, 693 datasets. **(E)** The ROC curve analysis for KRTCAP2 expression in glioma patients. **(F–H)** OS, DFI and DSS survival curve of KRTCAP2 mRNA in high and low expression groups. *P < 0.05, **P < 0.01. ***P < 0.001.

We first investigated KRTCAP2 mRNA expression in glioma compared to non-tumor brain tissues by integrating data from the TCGA and GTEx databases. Our analysis revealed a marked upregulation of KRTCAP2 transcription in glioma samples (**Figure 1A**). Consistent with this, KRTCAP2 expression in the TCGA cohort increased progressively from Grade II to Grade IV (**Figure 1B**). To further validate these findings, we analyzed KRTCAP2 mRNA expression using the CGGA database, which confirmed that higher WHO grades were associated with elevated KRTCAP2 expression levels (**Figures 1C, D**). KRTCAP2 may serve as a potential diagnostic biomarker, as indicated by an AUC of 0.984 (**Figure 1E**). Further analysis of patient outcomes indicated that elevated KRTCAP2 expression was significantly associated with poorer overall, disease-specific, and disease-free survival in glioma patients (**Figures 1F–H**). Collectively, these results highlight the clinical significance of KRTCAP2 and suggest its dual potential as both a diagnostic and prognostic biomarker in glioma.

### KRTCAP2 protein expression and clinicopathological characteristics in glioma patients

**Figure 2:**
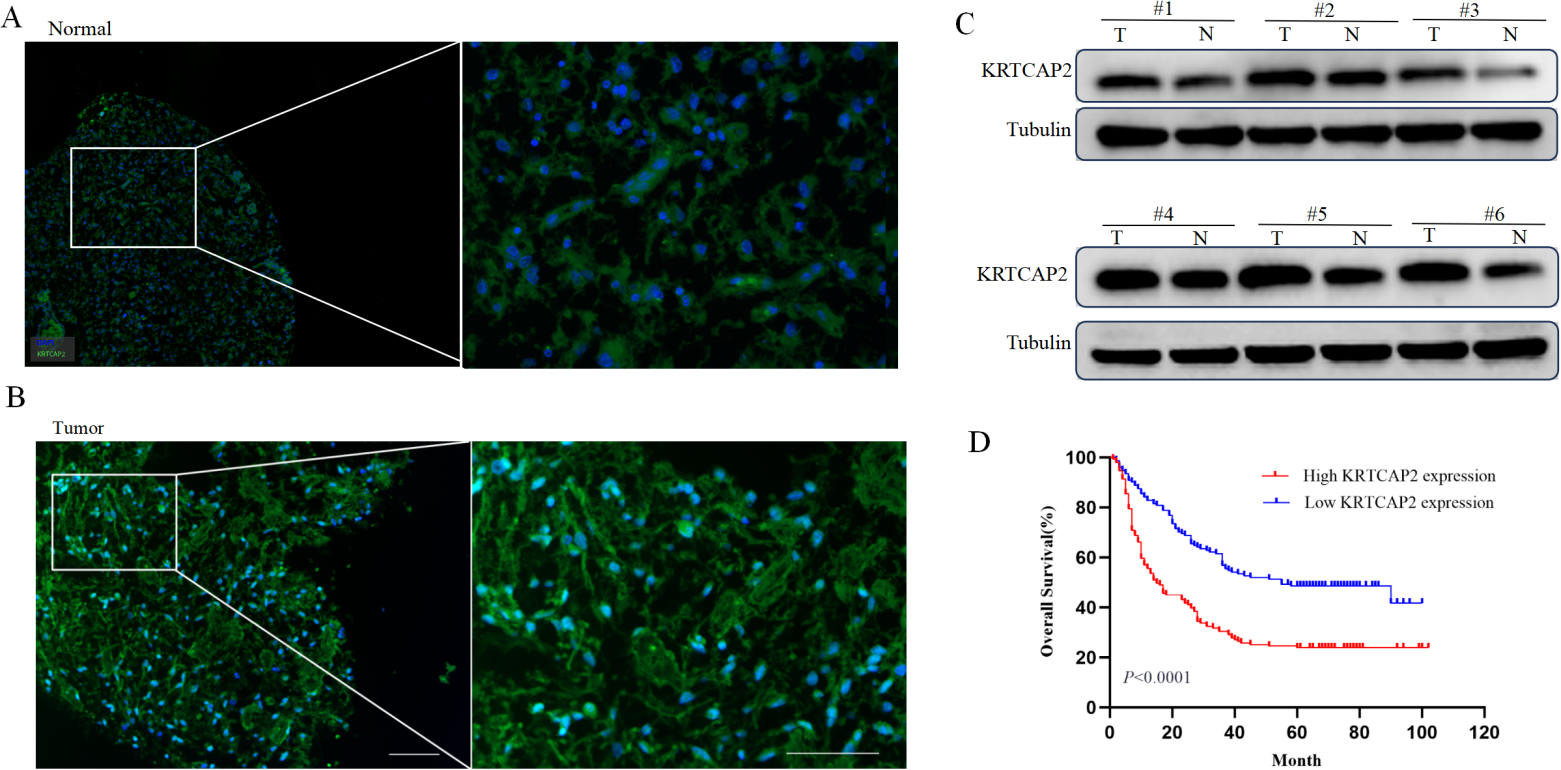
KRTCAP2 protein expression and clinicopathological characteristics of in glioma patients. **(A, B)** The representative multiplex-immunohistochemistry images of KRTCAP2 protein expression in clinical patient tissue microarrays. **(C)**KRTCAP2protein expression in six pairs of fresh surgical excision of glioma tissues (T) and adjacent non-tumor brain tissues. **(D)** The impacts of KRTCAP2 protein expression levels on the overall survival of glioma patients. Scale bar = 20 μm.

To investigate the potential roles of KRTCAP2 within the glioma TME, we first determined KRTCAP2 protein expression in patient TMAs using mIHC staining. Consistent with the mRNA expression shown above, KRTCAP2 protein was found more abundant in glioma tissue than in none tumor brain tissues (**Figure 2A, B**). Similarly, elevated KRTCAP2 protein expression was detected in glioma tissues compared to paired non-tumor brain tissues obtained from surgical resections (**Figure 2C**), This finding is consistent with the previously observed upregulation at the mRNA level and correlates with the malignant phenotype of glioblastoma.

To further assess the clinical relevance of KRTCAP2 protein expression, we analyzed its association with key clinicopathological parameters in glioma patients. Using an optimized cutoff value, the cohort was divided into groups with low/no and high KRTCAP2 expression. Statistical analysis revealed a marked association between the expression levels of KRTCAP2 and molecular subtype (Pearson x^2^ = 8.504, P = 0.003), as well as WHO tumor grade (Pearson x² =9.020, P = 0.011) (**Table 1**).

**Table 1:** Relationship between the expression of KRTCAP2 and clinicopathological characteristics in glioma patients.

Characteristic	N	KRTCAP2 expression			
		Low or no expression (%)	High expression (%)	Pearson χ^2^	*P* value
Total	303	127 (41.9)	176 (58.1)		
Gender				1.564	0.211
Male	171	77 (45.0)	82 (48.0)		
Female	132	50 (37.9)	82 (62.1)		
Age				2.289	0.130
<60	194	78 (40.2)	116 (59.8)		
≥60	109	49 (45.0)	60 (55.0)		
Histological type				6.497	0.09
a	134	64 (47.8)	70 (52.2)		
b	83	32 (38.6)	51 (61.4)		
c	30	7 (23.3)	23 (76.7)		
d	56	24 (42.9)	32 (57.1)		
Molecular type				8.504	0.003*
IDH1 ^R132H/mut^	244	92 (37.7)	152 (62.3)		
IDH1 ^R132H/WT^	59	35 (59.3)	24 (40.7)		
WHO grade				9.020	0.011*
I& II	97	29 (29.9)	68 (70.1)		
III	64	28 (43.8)	36 (56.3)		
IV	142	70 (49.3)	72 (50.7)		

*P < 0.05.

a, Astrocytoma, IDH1^R132H^ mutant. b, Oligodendroglioma, IDH1^R132H^ mutant. c, GBM, IDH1^R132H^ wild type. d, Pilocytic astrocytoma, pilomyxoid astrocytoma, and pleomorphic xanthoastrocytoma.

### Prognostic potential of KRTCAP2 protein expression in glioma patients

To identify prognostic factors influencing glioma patient outcomes, univariate and multivariate Cox regression analyses were performed. Univariate analysis indicated that KRTCAP2 protein expression (HR = 2.957, P < 0.001), age (HR = 0.592, P < 0.001), gender (HR = 0.667, P = 0.006), and WHO grade (HR = 1.954, P < 0.001) were significantly associated with overall survival. Subsequent multivariate analysis further established KRTCAP2 protein expression (HR = 4.683, P < 0.001) and WHO grade (HR = 2.363, P < 0.001) as independent prognostic factors for survival (**Table 2**). Consistent with these findings, Kaplan-Meier survival curves demonstrated that patients with high KRTCAP2 expression had significantly shorter overall survival (**Figure 2D**), underscoring its potential value as a prognostic biomarker in glioma.

**Table 2:** Univariate and multivariable analyses of prognostic factors for 5-year survival in glioma patients.

	Univariate analysis				Multivariate analysis			
	HR	*P* >|z|	95% CI		HR	*P* >|z|	95% CI	
KRTCAP2 expression High vs Low or none	2.957	<0.001*	2.158	4.052	4.683	<0.001*	3.367	6.514
Age (y) ≤ 60 vs > 60	0.592	<0.001*	0.443	0.787	0.846	0.288	0.622	1.152
Gender Male vs Female	0.667	0.006*	0.498	0.893	0.807	0.168	0.595	1.095
Histological type a vs. b vs. c vs. d	1.086	0.162	0.967	1.220				
Molecular type IDH1^R132H/mut^ vs IDH1^R132H/WT^	1.320	0.147	0.907	1.920				
WHO grade 1& 2 vs 3 vs 4	1.954	<0.001*	1.619	2.358	2.363	<0.001*	1.900	2.939
Chemotherapy TMZ vs None	1.275	0.222	0.863	1.883				
Radiotherapy Yes vs No	1.183	0.321	0.849	1.649				

*p < 0.05.

a, Astrocytoma, IDH1^R132H^ mutant. b, Oligodendroglioma, IDH1^R132H^ mutant. c, GBM, IDH1^R132H^ wild type. d, Pilocytic astrocytoma, pilomyxoid astrocytoma, and pleomorphic xanthoastrocytom.

### KRTCAP2 expression in glioma cells

**Figure 3:**
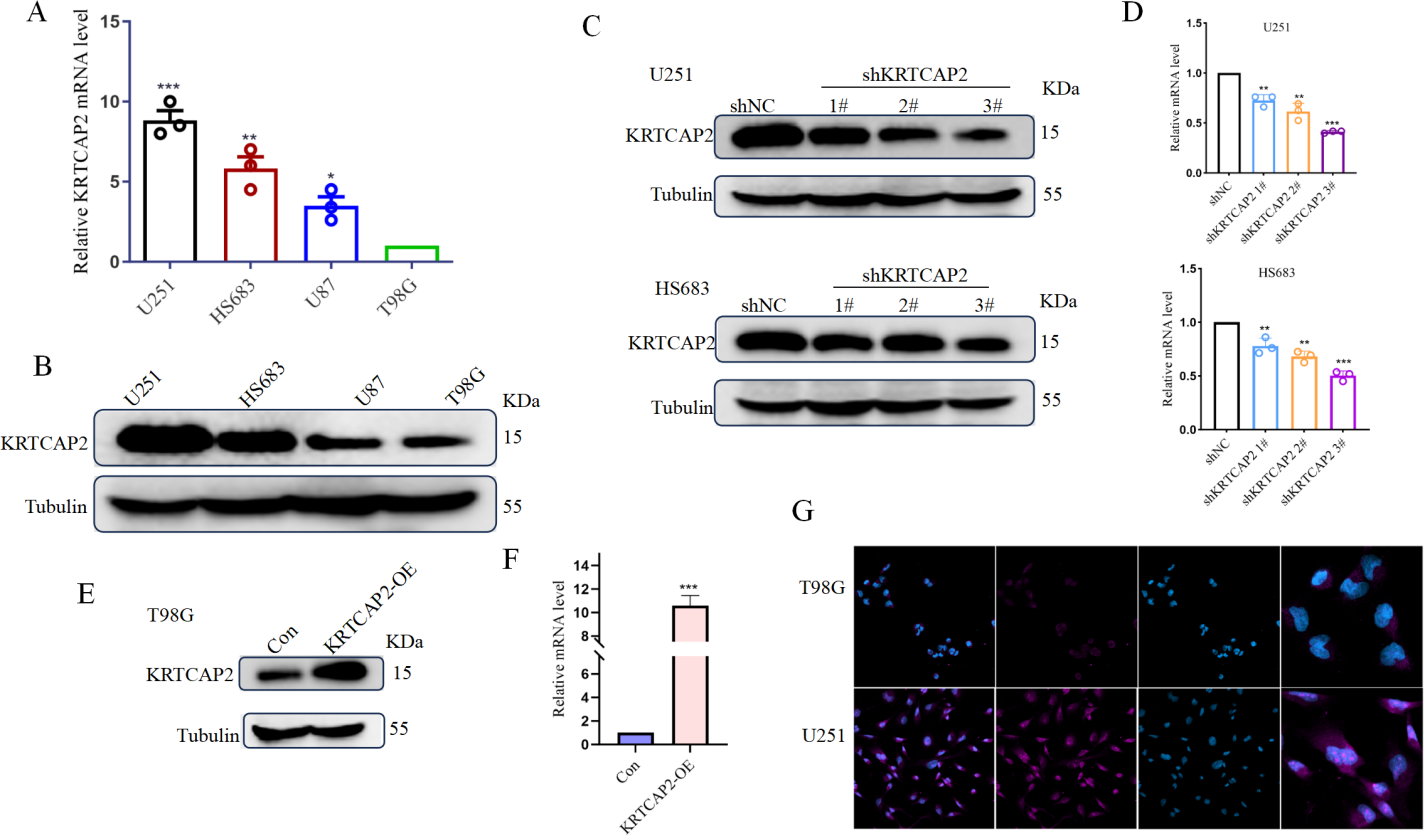
Expression of KRTCAP2 in glioma cell lines. **(A, B)** mRNA and protein expression of KRTCAP2 in glioma cell lines U251, HS683, U87, and T98G. **(C-F)** Western blot and RT-PCR analysis of KRTCAP2 expression in KRTCAP2-depleting U251 and HS683 cells and KRTCAP2 overexpressing T98G cells. Tubulin was used as the loading control. **(G)** Immunofluorescence images of KRTCAP2 (red) and DAPI (blue) staining in T98G and U251 cells (×200). *P < 0.05, **P < 0.01. ***P < 0.001.

To explore the biological roles and phenotypic impacts of KRTCAP2 in glioma, we first measured its expression in four glioma cell lines through qRT-PCR and western blot analyses. Results indicated that KRTCAP2 mRNA and protein levels were substantially elevated in U251 and HS683 cells relative to U87MG and T98G cells (**Figures 3A, B**). To further investigate the oncogenic potential of KRTCAP2, we selected U251 and HS683 cell lines, which exhibited higher KRTCAP2 expression, and transfected them with Lv-shNC or Lv-shKRTCAP2. In contrast, T98G cells, which displayed lower basal KRTCAP2 expression, were transfected with a KRTCAP2-expression vector. Transfection efficiency was validated by qRT-PCR and western blot, the results demonstrated that KRTCAP2 expression was significantly downregulated in U251 and HS683 cells after transfection with shRNA, especially shRNA3, and significantly upregulated in T98G cells after transfection with KRTCAP2-expression vector (**Figures 3C–F**). These findings were further confirmed by immunofluorescence experiments (**Figure 3G**).

### KRTCAP2 promotes glioma cell proliferation and metastasis *in vitro*

**Figure 4:**
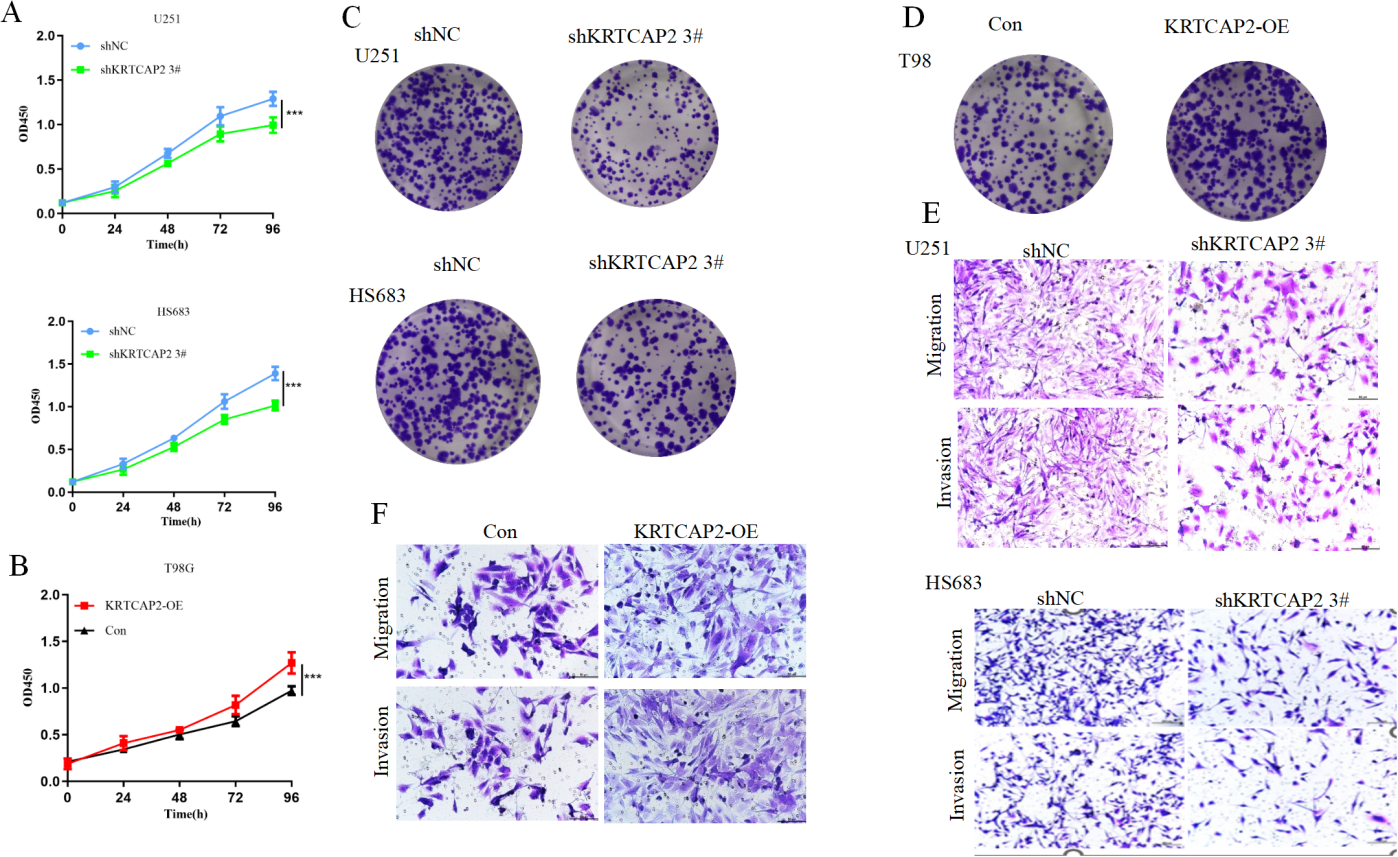
KRTCAP2 promotes glioma cell proliferation and metastasis *in vitro*. **(A, B)** The cells growth rates were determined by CCK-8 proliferation assays at various time points. **(C, D)** Representative images of colony formation assays are shown to display the proliferation ability of CRC cells after transfection. **(E, F)** Transwell analysis was preformed to determine the cell migration and invasion. ***P < 0.001.

To investigate the influences of KRTCAP2 on glioma cell proliferation, we conducted CCK-8 and colony-formation assays. The results indicated that knockdown of KRTCAP2 significantly suppressed cell growth in U251 and HS683 cells, whereas overexpression of KRTCAP2 enhanced cellular proliferation compared to the corresponding control groups (**Figures 4A–D**). We next evaluated whether KRTCAP2 plays a role in glioma metastasis by measuring its effects on cell migration and invasion. Transwell assays showed that downregulation of KRTCAP2 markedly attenuated both migration and invasion. Conversely, overexpression of KRTCAP2 significantly enhanced these malignant behaviors (**Figures 4E, F**). These findings indicated that KRTCAP2 modulates the biological functions of glioma cells.

### KRTCAP2 mRNA expression correlates with immune cell infiltration in glioma

**Figure 5:**
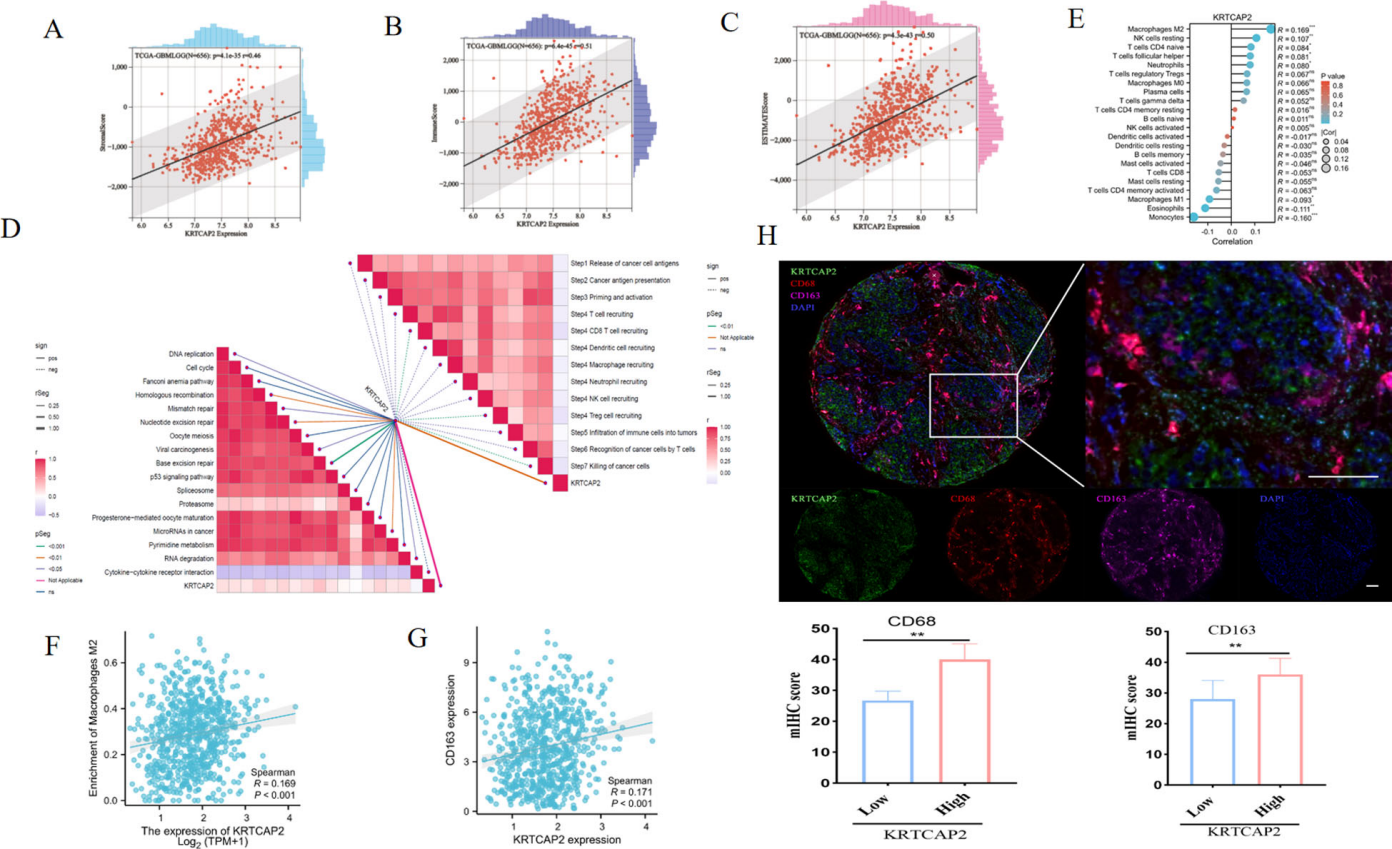
KRTCAP2 mRNA expression correlates with immune cell infiltration in glioma. **(A-C)** The correlation between KRTCAP2 mRNA expression and stromal, immune, and ESTIMATE score. **(D)** The Pearson correlation analysis showing the correlation of KRTCAP2 expression with multiple anti-cancer immune process and immunotherapy-related pathways based on GSVA score for individual patients in the TCGA database. GSVA, gene set variation analysis. **(E)** CIBERSORT analysis of 22 infiltrating immune cell types associated with KRTCAP2 in glioma. **(F, G)** Correlation of KRTCAP2 expression with M2 macrophages and CD163 expression. **(H)** Fluorescence-based mIHC staining showing spatial distribution and co-localization of KRTCAP2 and immune markers. KRTCAP2 (green), CD68 (red), CD163 (purple), and DAPI (blue). Scale bar, 20 μm. *p < 0.05, **P < 0.01, ***P < 0.001.

To elucidate the relationship between KRTCAP2 expression and the TME in glioma, we systematically evaluated KRTCAP2 levels in relation to glioma immune characteristics. Using the ESTIMATE algorithm, which quantifies stromal and immune components based on transcriptional profiles, we observed that higher KRTCAP2 expression correlated significantly with elevated stromal, immune, and combined ESTIMATE scores (**Figures 5A–C**), suggesting increased infiltration of non-tumor cellular components. Employing the ssGSEA algorithm, we computed the anti-cancer immune process matrix scores and immunotherapy-associated pathway enrichment values for each patient within the TCGA database. Pearson correlation analysis revealed that KRTCAP2 expression is significantly associated with multiple anti-cancer immune processes and immunotherapy-related pathways (**Figure 5D**).

Subsequently, we also examined the influence of KRTCAP2 on immune cell infiltration and found it positively associated with the abundance of M2 macrophages, CD4+ T cells, and neutrophil (**Figure 5E**). Additionally, KRTCAP2 displayed a positive correlation with immunosuppressive macrophage markers, including CD68 and CD163 (**Figures 5F, G**), suggesting that KRTCAP2-positive macrophages may constitute an immunosuppressive cell subset that facilitates the development of an immunosuppressive TME, promotes tumor immune escape, and ultimately accelerates tumorigenesis and progression. In order to validate the correlation between KRTCAP2 protein expression and immune cell abundance in the glioma immune microenvironment, we performed multiplex immunohistochemistry (mlHC) on glioma tissue microarrays. The results confirmed that KRTCAP2 protein expression was positively correlated with the density of CD68^+^ macrophages and CD68^+^CD163^+^ double-positive cells (**Figure 5H**). These findings indicate that CD163^+^ macrophages may contribute to glioma progression in a KRTCAP2-dependent expression.

### KRTCAP2 and drug responsiveness

**Figure 6:**
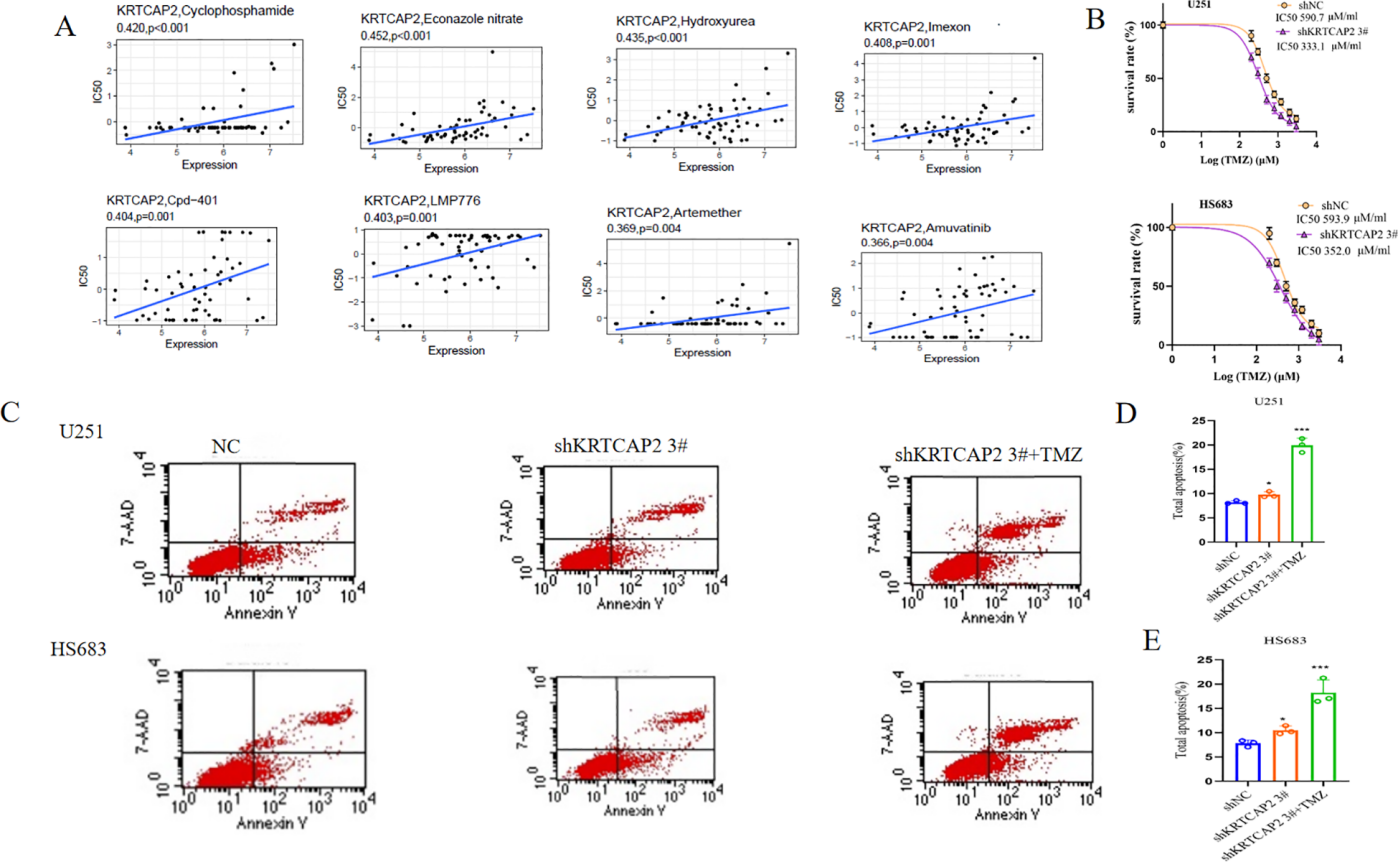
KRTCAP2 and drug responsiveness. **(A)** Analysis of the association between KRTCAP2 expression and predicted responsiveness to multiple therapeutic agents. **(B)** CCK8 analysis displays the cell survival rates after treatment with different TMZ concentrations in the U251 and HS683 cell lines. **(C, D)** Apoptosis assays demonstrating changes in glioma cell viability among the shNC, shKRTCAP2, and combined TMZ + shKRTCAP2 treatment groups. (*p < 0.05, ***P < 0.001).

KRTCAP2 expression was inversely related to drug responsiveness among patients treated with Cyclophosphamide, Econazole nitrate, Hydroxyurea, Imexon, Cpd-401, LMP776, Artemether, Amuvatinib. **Figure 6A** depicts the association between KRTCAP2 expression and predicted drug responsiveness. Furthermore, the percentage of early and total apoptotic cells was significantly higher in U251 and HS683 cells after KRTCAP2 knockdown compared with the shNC control group. Temozolomide (TMZ) is a second-generation oral alkylating agent and a first-line chemotherapy drug for gliomas, known for its ability to cross the blood-brain barrier, rapid and complete absorption, and high bioavailability, making it the standard treatment of glioblastoma multiforme (GBM) (24–26). We then treated U251 and HS683 cell lines with varying concentrations of TMZ and observed that the IC50 of TMZ decreased following KRTCAP2 knockdown. These findings suggest a correlation between KRTCAP2 expression and TMZ resistance in glioma (**Figure 6B**). Apoptosis analysis further revealed that temozolomide (TMZ) administration alone significantly reduced glioma cell viability, as did KRTCAP2 knockdown. Notably, the combined application of TMZ and KRTCAP2 silencing produced a stronger anti-proliferative outcome than either intervention alone, indicating a possible synergistic effect between TMZ chemotherapy and KRTCAP2 suppression (**Figure 6C**).

## Discussion

Gliomas represent the most common form of primary brain tumor, characterized by aggressive growth, frequent recurrence, and poor clinical outcomes. The current standard treatment involves maximal surgical resection followed by radiotherapy and chemotherapy (27, 28). However, due to factors such as the blood-brain barrier and the tumor microenvironment, challenges including radiation resistance, limited chemotherapeutic drugs efficacy, and chemoresistance frequently arise, ultimately leading to suboptimal treatment outcomes and very unfavorable long-term prognosis. Given the current limitations in glioma treatment, it is imperative to understand glioma development, investigate key differential genes, and identify novel therapeutic targets to improve patient survival.

Recent studies have established that KRTCAP2 activation is a common feature across multiple cancers, contributing to tumor progression by modulating processes such as cell adhesion, invasion, and immune evasion. For example, KRTCAP2 expression is significantly correlated with pathological features of hepatocellular carcinoma, including clinical stage and degree of differentiation, and patients with high KRTCAP2 levels exhibit a higher risk of poor prognosis (29, 30). In this study, we utilized bioinformatics methods to examine the relationship between KRTCAP2 mRNA expression and clinical outcomes in glioma patients, as well as its association with clinicopathological features. Our results demonstrated that both KRTCAP2 mRNA and protein levels were significantly elevated in glioblastoma (GBM) tissues compared to adjacent non-tumor tissues. Elevated KRTCAP2 protein expression levels were associated with more advanced TNM stages, implying a potential involvement in tumor progression. According to Kaplan-Meier survival curves, patients exhibiting low KRTCAP2 expression experienced markedly longer survival, underscoring its utility as a prognostic biomarker in GBM. Complementary *in vitro* experiments further demonstrated that KRTCAP2 augments proliferation, migration, and invasion in glioma cell, suggesting its contribution to gliomagenesis through the promotion of these oncogenic phenotypes.

Immune cells are a critical component of the glioma TME, and their spatial distribution, infiltration abundance, cellular properties, and functional phenotypes can significantly influence the tumor’s biological behavior. These immune cells interact in diverse ways, either sustaining or promoting tumor proliferation and invasion while limiting the efficacy of most current anti-tumor therapies (31–33). To gain insights into the infiltration of immune cells within the TME, immune, stromal and ESTIMATE scores have been introduced by analyzing gene expression data from tumor samples. Our findings indicate that KRTCAP2 mRNA expression is associated with immune subtype classification, stromal score, and ESTIMATE score. Previous research have suggested that N-glycosylation mediated by KRTCAP2 may regulate immune cell proliferation and influence tumor cell adhesion and invasion processes (34). Huang et al. found KRTCAP2 expression showed significant correlations with infiltrating CD8^+^ and CD4^+^T cells (12). Macrophages are an essential component of immune infiltration in the glioma TME, with M2 macrophages promoting glioma progression and growth (35–38). Upregulation of KRTCAP2 contributes to the proliferation, migration, and invasiveness of glioma cells. Using mIHC, we analyzed d the spatial distribution and co-localization of KRTCAP2 with immune cell populations in the glioma TME. These findings indicate a significant association between KRTCAP2 and the TME, warranting further exploration.

Analysis using CellMiner revealed that KRTCAP2 expression is negatively correlated with the responsiveness to multiple therapeutic drugs. This drug resistance may be linked to KRTCAP2’s role in DNA damage repair. Temozolomide (TMZ) is the standard chemotherapeutic agent for glioma, capable of crossing the blood-brain barrier to target cancer cells. Studies have shown that TMZ can inhibits proliferation, migration, and invasion of glioma cells by suppressing the MEK/ERK signaling pathway, and reducing vascular endothelial growth factor (VEGF) expression (39). CCK8 and apoptosis assays further demonstrated that both TMZ treatment and KRTCAP2 knockout independently promote apoptosis in glioma cells. Notably, the combined application of TMZ and KRTCAP2 silencing produced a stronger pro-apoptotic effect, suggesting a potential synergistic interaction in suppressing glioma cell growth. These findings indicate that targeting KRTCAP2 could represent a promising strategy to enhance chemotherapy efficacy in glioma treatment.

Nonetheless, this study has several limitations. First, additional evidence from animal models is needed to further elucidate the role of KRTCAP2 in glioma progression and macrophage polarization. Second, although we observed a correlation with clinical outcomes, the underlying mechanisms remain unclear. Furthermore, the current dataset is cross-sectional and lacks longitudinal validation.

## Conclusions

In summary, our results indicate that aberrant KRTCAP2 expression is an independent prognostic predictor in glioma. Furthermore, we provide evidence that KRTCAP2 directly promotes malignant progression. Taken together, KRTCAP2 represents a promising dual-purpose candidate, serving both as a prognostic biomarker and a therapeutic target for immune-based strategies, with the potential to improve clinical outcomes in glioma patients.

## Data Availability

The datasets presented in this study can be found in online repositories. The names of the repository/repositories and accession number(s) can be found in the article/supplementary material.
